# Pre-exposure prophylaxis with tixagevimab/cilgavimab (AZD7442) prevents severe SARS-CoV-2 infection in recipients of allogeneic hematopoietic stem cell transplantation during the Omicron wave: a multicentric retrospective study of SFGM-TC

**DOI:** 10.1186/s13045-022-01387-0

**Published:** 2022-11-28

**Authors:** Ludovic Jondreville, Maud D’Aveni, Hélène Labussière-Wallet, Amandine Le Bourgeois, Alban Villate, Ana Berceanu, Silvia-Maria Bezsera, Anne Thiebaut, Marion Boissard-Simonet, Marlène Legrand, Jérôme Cornillon, Marie-Thérèse Rubio, Patrice Chevallier, Stéphanie Nguyen

**Affiliations:** 1 Department of Clinical Hematology, Groupe Hospitalier Pitié-Salpêtrière, AP-HP Sorbonne Université, 47-83 Boulevard de L’Hôpital, 75013 Paris, France; 2 Department of Clinical Hematology, Centre Hospitalier Régional Universitaire de Nancy, Vandoeuvre-lès-Nancy, France; 3 Department of Clinical Hematology, Centre Hospitalier Lyon-Sud, Hospices Civils de Lyon, Pierre-Bénite, France; 4 Department of Clinical Hematology, Nantes University Hospital, Nantes, France; 5 Department of Clinical Hematology, Centre Hospitalier Régional Universitaire de Tours, Tours, France; 6 Department of Clinical Hematology, Besançon University Hospital, Besançon, France; 7 Institut de Cancérologie Lucien Neuwirth, Saint-Priest-en-Jarez, France; 8 Department of Clinical Hematology, Centre Hospitalier Universitaire Grenoble-Alpes, Grenoble, France

**Keywords:** SARS-CoV-2, Allogeneic stem cell transplantation, Pre-exposure prophylaxis, AZD7442

## Abstract

Since the emergence of the Omicron variant of SARS-CoV-2, though considered less virulent, hospitalization and death rates among immunocompromised patients remain high, especially for poor responders to vaccination. We conducted a retrospective multicentric study to evaluate pre-exposure prophylaxis with AZD7442 (tixagevimab/cilgavimab) for preventing COVID-19 in adult allogeneic hematopoietic stem cell transplant (allo-HSCT) recipients. Among the 161 patients of our cohort, 22 (14%) contracted COVID-19 after a median follow-up of 105 days, but no severe form was observed. Only one major adverse event was reported: an acute coronary syndrome, resolved without sequelae. Pending randomized controlled trial results, our data support the use of AZD7442 as pre-exposure prophylaxis for COVID-19 during Omicron wave in allo-HSCT patients who failed to develop humoral immunity to vaccination, to prevent severe and potentially lethal forms of SARS-CoV-2 infection.

**Supplementary Information:**

The online version contains supplementary material available at 10.1186/s13045-022-01387-0.

To the Editor,

Recipients of allogeneic hematopoietic stem cell transplant (allo-HSCT) have a higher risk of severe form of coronavirus disease 2019 (COVID-19) infection than the general population [1].

The Omicron variant of SARS-CoV-2 has emerged in November 2021 and rapidly become the most dominant variant of concern worldwide [2, 3]. To date, few data are available about the severity of Omicron sublineages in immunocompromised patients, but the rates of hospitalization and mortality remain significant—about 20% and 9–13%, respectively—even for vaccinated patients [4, 5].

Despite reinforced vaccination schemes, about 20% of allo-HSCT patients fail to develop a robust humoral response. For these vulnerable patients, the use of tixagevimab/cilgavimab (AZD7442, AstraZeneca, Evusheld®) has been approved for pre-exposure prophylaxis against SARS-CoV-2, on the basis of the results of PROVENT study [6]. However, this study included very few immunocompromised patients and was conducted before the emergence of Omicron variant.

In our retrospective multicentric study, we aimed at assessing the incidence and severity of SARS-CoV-2 infections among allo-HSCT patients who received AZD7442 for pre-exposure prophylaxis.

AZD7442 was provided through a compassionate-use program, with eligibility criteria including anti-SARS-CoV-2-spike IgG titers < 260 binding antibody units (BAU)/mL and a negative test for SARS-CoV-2. Between December 2021 and April 2022, all allo-HSCT patients from participating centers who met the inclusion criteria and received AZD7442 were enrolled, unless they were already participating in a prospective study. All patients gave written consent prior to transplant for their data to be collected and used for research purposes, in accordance with the Declaration of Helsinki. This study was approved by the SFGM-TC scientific council.

One hundred and sixty-one patients from 8 HSCT centers, with a median age of 58 years old (range, 21–74), were enrolled (Additional file 1: Table S1 and Additional file 2: Table S2). The median period between allo-HSCT and AZD7442 administration was 289 days (interquartile range [IQR] 107–552). At inclusion, 117 patients (73%) had received up to four doses of SARS-CoV-2 vaccine. At least one factor of poor response to vaccination was found in 141 (88%) of our patients (Table 1). All patients received 300-mg AZD7442 (tixagevimab and cilgavimab, 150 mg each) in accordance with the guidelines at the time of this study. AZD7442 was generally well-tolerated, with only one major adverse event reported in a patient who experienced acute coronary syndrome (see Additional file 3: Clinical report and Additional file 4: Table S3).

**Table 1:** Baseline characteristics of the patients

	Symptomatic SARS-CoV-2 infection			
	Overall	Uninfected	Infected	*P* value*
	*N* = 161	*N* = 139	*N* = 22	
Median age at inclusion (range)—years	57.7 (21–73.9)	58 (21–73.9)	50.3 (29.3–72.7)	0.18
≥ 50 years old—*n* (%)	112 (70)	100 (72)	12 (55)	
< 50 years old—*n* (%)	49 (30)	39 (28)	10 (45)	
Number of vaccine doses prior to inclusion—*n* (%)				
4 doses	19 (12)	17 (12)	2 (9.1)	0.10
3 doses	71 (44)	61 (44)	10 (45)	
2 doses	22 (14)	15 (11)	7 (32)	
1 dose	5 (3.1)	5 (3.6)	0 (0)	
0 dose	44 (27)	41 (29)	3 (14)	
Prior history of SARS-CoV2 infection—*n* (%)^†^	13 (10)	11 (10)	2 (11)	> 0.99
Median anti-SARS-CoV-2-spike IgG titer (IQR)—BAU/mL^†^	16 (1–91)	16 (1–88)	15 (4–98)	0.93
Median time between allo-HSCT and inclusion (IQR)—days	289 (107–552)	302 (112–560)	275 (100–529)	0.80
< 12 months—*n* (%)	97 (60)	84 (60)	13 (59)	0.90
Ongoing immunosuppressive treatment—*n* (%)^†^	100 (62)	83 (60)	17 (77)	0.12
History of GvHD requiring systemic treatment—*n* (%)^†^	52 (44)	46 (45)	6 (40)	0.71
Median absolute lymphocyte count (IQR)^†^	730 (375–1360)	701 (368–1272)	1370 (495–1835)	0.12
< 1.0 G/L—*n* (%)	81 (60)	72 (62)	9 (47)	0.23
Median CD4+ T-cell count (IQR)^†^	116 (79–238)	113 (80–227)	133 (74–354)	0.42
< 500/mm^3^—*n* (%)	111 (94)	97 (94)	14 (93)	> 0.99
< 200/mm^3^—*n* (%)	75 (64)	67 (65)	8 (53)	0.38
Median CD19+ B-cell count (IQR)^†^	15 (0–112)	33 (0–135)	0 (0–46)	0.057
< 100/mm^3^—*n* (%)	68 (70)	55 (66)	13 (93)	0.058
< 70/mm^3^—*n* (%)	58 (60)	47 (57)	11 (79)	0.12
Median gamma-globulin level (IQR)^†^	5.6 (4.0–7.3)	5.6 (3.8–7.2)	5.8 (4.4–7.3)	0.67
< 7.0 g/L—*n* (%)	93 (71)	81 (72)	12 (67)	0.66
< 5.0 g/L—*n* (%)	47 (36)	41 (36)	6 (33)	0.81

*SARS-CoV-2* severe acute respiratory syndrome coronavirus 2, *IQR* interquartile range, *BAU* binding antibody unit, *HSCT* hematopoietic stem cell transplantation, *GvHD* graft-versus-host disease

*Using Fisher's exact test, Wilcoxon rank sum test or Pearson's Chi-squared test, as appropriate

^†^Missing data: 32 for history of SARS-CoV-2 infection; 10 for anti-SARS-CoV-2-spike IgG titer; 1 for ongoing immunosuppressive treatment; 44 for history of significant GvHD; 26 for absolute lymphocyte count; 43 for CD4+ T-cell count; 64 for CD19+ B-cell count; 20 for gamma globulins

The observation period for the study spanned from December 2021 to June 2022, while Omicron was reported as by far the most prevalent variant in this period of time in France and worldwide [2, 7]. With a median follow-up of 105 days (IQR, 82–119) after the injection of AZD7442, 22 out of 161 patients (14%) developed a symptomatic SARS-CoV-2 infection. The cumulative incidence of infection post-AZD7442 was 8% (95% CI 3–13) at 30 days and 16% (95% CI 7–24) at 90 days (Fig. 1). For these 22 patients, the median time between AZD7442 and infection was 33.5 days. No reliable predictive factors of SARS-CoV-2 infection were found (Table 1). No severe COVID-19 requiring hospitalization was encountered. Eight patients out of 22 (44%) received an additional treatment in the days following the infection (sotrovimab, *N* = 4; nirmatrelvir–ritonavir, *N* = 3; tixagevimab–cilgavimab, *N* = 1). Overall, no patient died from SARS-CoV-2 infection.

**Fig. 1:**
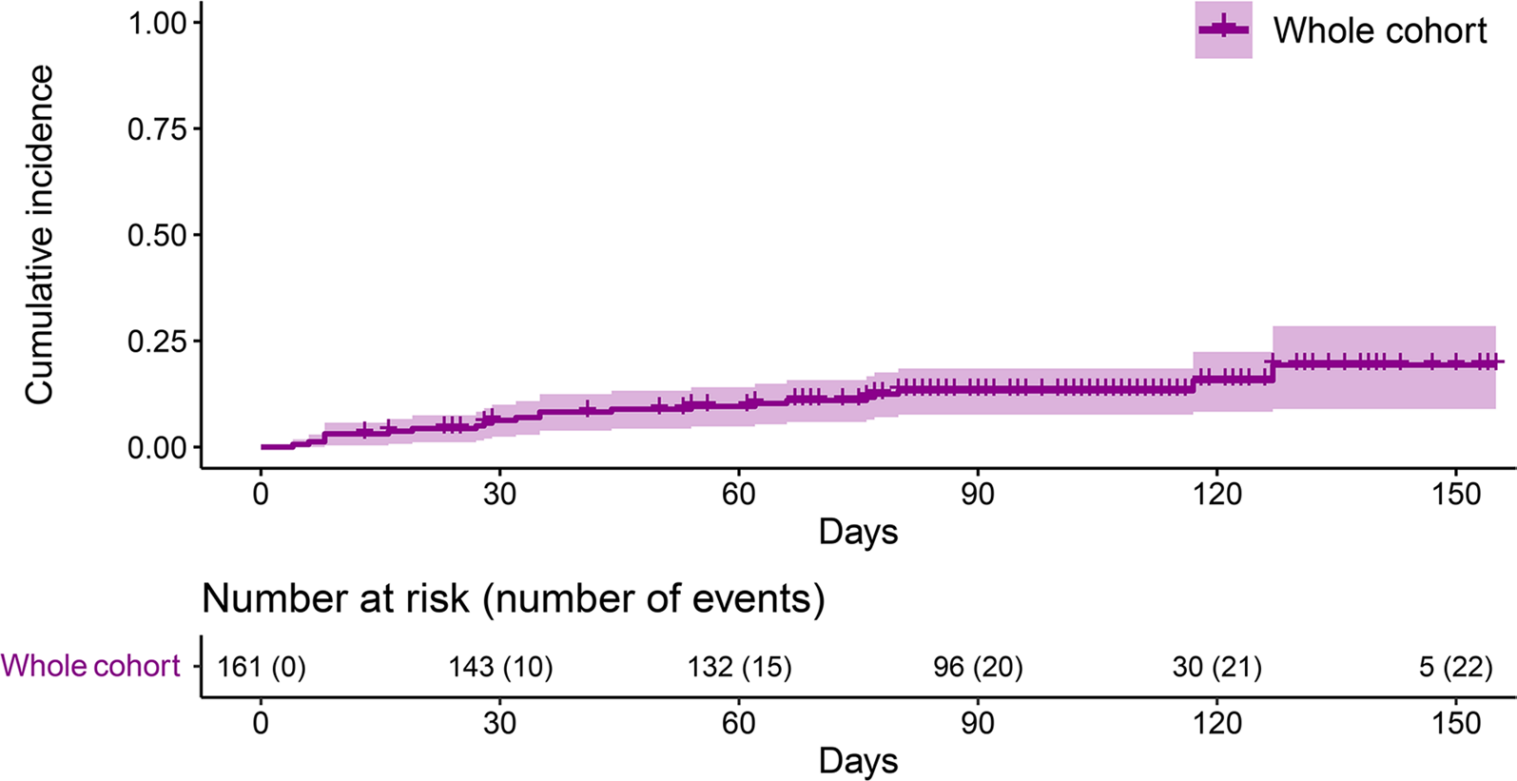
Cumulative incidence of SARS-CoV-2 infection after administration of AZD7442

Four recent studies on immunocompromised patients receiving AZD7442 during the same period showed an average incidence rate of 4% (3.5–5) for COVID-19 [4, 5, 8, 9]. Two of them had a control group, against which the AZD7442 group compared favorably (5% vs. 14% and 3.5% vs. 7.2%, respectively, *P* < 0.001 each) [4, 5]. Another study in kidney transplant recipients suggested that AZD7442 could not prevent severe form of COVID-19 in these patients (36% hospitalized, including 8% in ICU; 5% died), with a 300-mg dose [10]. These data are summarized in Additional file 5: Table S4.

Interestingly, two of these studies found that a dose escalation to 600 mg led to a lower incidence rate of COVID-19. The TACKLE study demonstrated that 600-mg AZD7442 was safe and significantly reduced the odds of progression to severe disease in COVID-19 patients [11]. Therefore, the 600-mg dose has become the recommended dose in pre-exposition in France since July 2022. Few data are currently available on the neutralizing capacity of sera from AZD7442-treated patients for Omicron variant [12]. A prospective study is ongoing to validate this strategy and assess the impact of this dose escalation on the neutralizing antibody activity in patients’ sera (PRECOVIM clinical trial, NCT05216588).

## Supplementary Information

**Additional file 1: Table S1.** Characteristics of allo-HSCT.

**Additional file 2: Table S2.** Underlying comorbidities at the time of AZD7442 administration.

**Additional file 3: Clinical report.** Serious adverse event: acute coronary syndrome.

**Additional file 4: Table S3.** Adverse events.

**Additional file 5: Table S4.** Comparison of incidence rate of COVID-19 during Omicron wave.

## Data Availability

All data generated or analyzed during this study are included in this published article and its supplementary information files.

## Abbreviations

Allo-HSCT: Allogeneic hematopoietic stem cell transplant
COVID-19: Coronavirus disease 2019
SARS-CoV-2: Severe acute respiratory syndrome coronavirus 2
BAU: Binding antibody units
IQR: Interquartile range
