# Supplementary material for: Pre-exposure prophylaxis with tixagevimab/cilgavimab (AZD7442) prevents severe SARS-CoV-2 infection in recipients of allogeneic hematopoietic stem cell transplantation during the Omicron wave: a multicentric retrospective study of SFGM-TC
Source: J Hematol Oncol. 2022 Nov 28;15:169. doi: 10.1186/s13045-022-01387-0 (PMC9702670; doi:10.1186/s13045-022-01387-0)
Supplement: Supplementary file 3 — Additional file 3: Clinical report. Serious adverse event: acute coronary syndrome. [file 13045_2022_1387_MOESM3_ESM.docx]

ADDITIONAL FILE 3

**Serious adverse event: acute coronary syndrome.**

Only one patient of our cohort (1/161, 0.6%) encountered a serious adverse event in the form of acute coronary syndrome. This 62-year-old man underwent allo-HSCT one year before, in January 2021. He received 3 doses of mRNA Covid-19 vaccine at M5, M6 and M8 post-HSCT, but failed to develop a post-vaccination humoral response. Apart from age and sex, he had a 60 pack-years smoking history, quit 2 years ago; no other cardiovascular disease risk factor was reported. Therefore, he was given AZD7442 at M12, providing him with IgG anti-spike titer above 2080 BAU/mL one month later.

This patient felt chest pain 11 days after AZD7442 administration. The diagnosis of acute coronary syndrome was confirmed. The coronary angiography showed a significant stenosis of the right coronary artery (>90%) and distal left circumflex artery (50-70%), treated with stent implantation followed with cardiac rehabilitation. The patient recovered without sequelae, and the cardiac stress test performed 3 months later was normal.

In the PROVENT study (Levin et al. N Engl J Med 2022), 7 serious cardiac adverse events were reported, including acute left ventricular failure, acute myocardial infarction, and paroxysmal atrioventricular block, with a frequency of 0.2% (6/3461) in the AZD7442 arm, and 0.1% (1/1736) in the placebo arm (non-significant difference).

Within our study, it is not possible to draw any firm conclusion from an isolated case. The occurrence of acute coronary syndrome prompts caution, although the link with AZD7442 cannot be positively stated. The cardiovascular risk should be properly assessed in all patients before considering this treatment, and patients should be advised to report any evocative symptoms.
